# S03-1 Physical literacy, physical activity and wellbeing in Danish children

**DOI:** 10.1093/eurpub/ckac093.012

**Published:** 2022-08-29

**Authors:** Paulina Sander Melby, Glen Nielsen, Jan Christian Brønd, Peter Bentsen, Peter Elsborg

**Affiliations:** 1 Department of Nutrition, Exercise and Sports, University of Copenhagen; 2 Danish School sports; 3 Centre of Research in Childhood Health, Institute for Sport Science and Clinical Biomechanics, University of Southern Denmark, Odense, Denmark; 4 Center for Clinical Research and Prevention, Copenhagen University Hospital – Bispebjerg and Frederiksberg; 5 Health promotion research, Steno Diabetes Center Copenhagen; 6 Department of Geosciences and Natural Resource Management, University of Copenhagen, Copenhagen, Denmark

**Keywords:** Physical activity, physical literacy, well-being

## Abstract

**Background:**

PL is increasingly regarded as a ‘cause of the causes’ to health promotion. Cross-sectional studies have shown associations between children’s PL, physical activity (PA) behaviors and well-being. This presentation aims to present empirical evidence from a large-scale research project in Denmark assessing children’s physical literacy. Results from two studies will be presented and discussed. The first aims to examine the associations between Danish children’s PL and their physical and psychosocial well-being and whether the associations are mediated by moderate-to-high intensity physical activity (MVPA). The second aim is to examine the synergies between movement behaviors and levels of PL.

**Methods:**

Cross-sectional data from Danish school children aged 7-13 years were collected in Jan-Dec 2020 in the Danish Assessment of Physical Literacy (DAPL) project. PL was assessed with DAPL which measures the affective, cognitive, and physical domains of PL. Sedentary time and time spent in different PA intensities was measured with accelerometers (Axivity), psychosocial well-being was measured with The Strengths and Difficulties Questionnaire (SDQ), and physical wellbeing was measured with the KIDSCREEN questionnaire. In study 1, structural equation models were constructed with PL and MVPA as predictors of physical well-being and four aspects of psychosocial well-being. In study 2 a compositional analysis was applied with average weekly time spent sedentary, in low PA, moderate PA and vigorous PA as the compositional response and levels of PL as predictor.

**Results:**

A positive moderate association between PL and physical well-being, partly mediated by MVPA was observed. PL was positively associated with the positive aspects of psychosocial wellbeing and negatively associated with the negative aspects (behavior problems). None of the associations between PL and aspects of psychosocial wellbeing were mediated by MVPA. Results from study 2 are ongoing.

**Conclusions:**

The studies contribute to evidence on the link between PL, physical activity, and health outcomes. The study found beneficial relations between PL and physical and psychosocial well-being.

